# Key Note: “Health, Nutrition, Fitness and Wellbeing”, a definite current challenge

**Published:** 2014

**Authors:** Purcărea Theodor Valentin

**Affiliations:** * President of the Romanian Distribution Committee, Professor at the Romanian-American University, Member of the Scientific Committee of SANABUNA International Congress

**Abstract**

There is no doubt that the citizen’s quality of life may be improved by the right understanding of this definite current challenge of “Health, Nutrition, Fitness and Wellbeing” (SANABUNA), by continuously reflecting, staying optimistic, being realistic, applying the knowledge with wisdom, strengthening all this supply chain seen from the perspective of a trust-based “physician”-“patient” relation, managing the evidence accordingly and sending a consistent SANABUNA message to society. SANABUNA social movement must lead all stakeholders beyond the fragments of understanding, interacting, getting involved, communicating and learning how to realize the proper change of our behavior requiring a new thinking, a new policy, a proper education located in the heart of adaptation, and proving solidarity in building this trust-based “physician”-“patient” relation. There is no more doubt that nutrition represents the bridge between agriculture and health, and food is one of the greatest contemporary actors on the political scene, as well as the fact that public health aspects are often marginalized amid the competing interests of producers, processors, wholesalers, retailers, caterers and consumers. We are all consumers and patients and we all know that we have a serious problem, and we all know that there are no simple solutions. That is why there is an urgent need for more multidisciplinary, interdisciplinary and transdisciplinary research on food, nutrition and health, breaking down the “silos” between the sectors, considering relevant disciplines such as: agricultural research, economics, and policy; anthropology of food; physiology of food and fluid intake; nutrition and food habits and choices; sociology of diet, food and nutrition; psychology of food and nutrition; food marketing; consumer research; medicine and health; health education; public health; social services and public welfare; supply, demand and public policy. There are substantial differences regarding diet and health, and less substantial differences in the way food functions in the minds and lives of people. There are also differences in the consumers’ experiences and perceptions of food security, self-sufficiency, and of the link between food, nutrition, and health. Beyond the reality that agriculture is the main source of food to meet the consumers’ need for energy and essential nutrients and given that all along the agricultural value chain, there are opportunities to improve nutrition and reduce health risks, we need to look at food systems, considering all the stages from field to fork, starting from the determination of the place where value for nutrition can be integrated, by using risk analysis along the food value chain, and by proving a renewing commitment to nutrition education, which makes a difference in making healthy eating choices, integrating agriculture, nutrition, and health services, which involves focusing the academic research on reflecting both the way the links between agriculture, nutrition and health work, and the adequate way of utilizing the sets of tools that could help to leverage agriculture for better nutrition and health, while continuing to prove the correlation between nutrition and cognitive function and academic performance. We also need to understand and deal with the challenge of considering the integration of health and wellness dimensions, realizing that wellness is a product of a healthy lifestyle, taking into account the contribution of our physical activity to (physical, health-related physical, skill-related physical) fitness as the state in our health characteristics and behavior, as the state affecting physical, mental, and social health. However, can we all stakeholders in the continuum of research shape together the future of our “Health, Nutrition, Fitness and Wellbeing” and proactively go after it? How can the reflection of our networking together encourage SANABUNA social movement?

**Keywords:**health, nutrition, fitness, wellbeing

